# One health systems strengthening in countries: Tripartite tools and approaches at the human-animal-environment interface

**DOI:** 10.1136/bmjgh-2022-011236

**Published:** 2023-01-26

**Authors:** Stephane de la Rocque, Kaylee Marie Myhre Errecaborde, Guillaume Belot, Tianna Brand, Sean Shadomy, Sophie von Dobschuetz, Ryan Aguanno, Maud Carron, Francois Caya, Shanlong Ding, Madhur Dhingra, Daniel Donachie, Gyanendra Gongal, Peter Hoejskov, Gunel Ismayilova, Gael Lamielle, Heba Mahrous, Mariana Marrana, Serge Nzietchueng, Yooni Oh, Julio Pinto, Xavier Roche, Ana Riviere-Cinnamond, Cristina Rojo, Lisa Scheuermann, Julie Sinclair, Junxia Song, Artem Skrypnyk, Tieble Traore, Kachen Wongsathapornchai

**Affiliations:** 1 World Health Organization Headquarters, Geneva, Switzerland; 2 World Organisation for Animal Health Headquarters, Paris, France; 3 Food and Agriculture Organization of the United Nations Headquarters, Rome, Italy; 4 World Health Organization Regional Office for South-East Asia, New Delhi, Delhi, India; 5 World Health Organization Regional Office for Europe, Copenhagen, Denmark; 6 World Health Organization Regional Office for the Eastern Mediterranean, Cairo, Egypt; 7 Food and Agriculture Organization of the United Nations, Kenya Representation, Nairobi, Kenya; 8 Food and Agriculture Organization of the United Nations, Regional Office for Asia and the Pacific, Bangkok, Thailand; 9 Pan American Health Organization / World Health Organization Regional Office for the Americas, Washington, District of Columbia, USA; 10 Regional Office for Africa, World Health Organization Regional Office for Africa, Dakar-Hub, Dakar, Senegal

**Keywords:** COVID-19, Health policy, Health systems, Health systems evaluation, Public Health

## Abstract

Unexpected pathogen transmission between animals, humans and their shared environments can impact all aspects of society. The Tripartite organisations—the Food and Agriculture Organization of the United Nations (FAO), the World Health Organization (WHO), and the World Organisation for Animal Health (WOAH)—have been collaborating for over two decades. The inclusion of the United Nations Environment Program (UNEP) with the Tripartite, forming the ‘Quadripartite’ in 2021, creates a new and important avenue to engage environment sectors in the development of additional tools and resources for One Health coordination and improved health security globally. Beginning formally in 2010, the Tripartite set out strategic directions for the coordination of global activities to address health risks at the human-animal-environment interface. This paper highlights the historical background of this collaboration in the specific area of health security, using country examples to demonstrate lessons learnt and the evolution and pairing of Tripartite programmes and processes to jointly develop and deliver capacity strengthening tools to countries and strengthen performance for iterative evaluations. Evaluation frameworks, such as the International Health Regulations (IHR) Monitoring and Evaluation Framework, the WOAH Performance of Veterinary Services (PVS) Pathway and the FAO multisectoral evaluation tools for epidemiology and surveillance, support a shared global vision for health security, ultimately serving to inform decision making and provide a systematic approach for improved One Health capacity strengthening in countries. Supported by the IHR-PVS National Bridging Workshops and the development of the Tripartite Zoonoses Guide and related operational tools, the Tripartite and now Quadripartite, are working alongside countries to address critical gaps at the human-animal-environment interface.

**SUMMARY BOX**

- The crisis brought on by the COVID-19 pandemic demonstrates how pathogen transmission between animals, humans and their shared environment can impact all aspects of society, highlighting how response in one country has the potential to impact health systems globally.
- The Tripartite organisations recognise the need for a shared global vision for capacity strengthening at the human-animal-environment interface and have set out to provide a coordinated approach that can be adopted by all countries.
- Summarising lessons learnt from the implementation of successive and complementary assessments and activities in countries, a stepwise method is proposed by the Tripartite to support countries to organise and prioritise actions in critical technical areas at the human-animal-environment interface.
- Using Tripartite evaluation frameworks such as WHO International Health Regulations Monitoring and Evaluation Framework and the WOAH Performance of Veterinary Services Pathway, countries can cross-map respective sectoral needs and create a shared vision for multisectoral coordination.
- With the inclusion of UNEP in the Quadripartite, opportunities for strengthening health security through inclusion of the environment sector are outlined and promoted.

## Introduction

The global crisis brought on by the COVID-19 pandemic demonstrates how pathogen transmission between animals, humans and their shared environment can impact all aspects of society. Coordination between sectors, primarily human, animal and environment health, is critical for timely and effective preparedness and response measures in countries.^1^ As such, countries welcome guidance and benefit from extensive global partnership to operationalise many multisectoral, ‘One Health’ approaches needed for the identification and management of emerging, re-emerging and endemic health threats.^2–4^ The Tripartite–the Food and Agriculture Organization of the United Nations (FAO), the World Health Organization (WHO), and the World Organisation for Animal Health (WOAH)–operationalised their collaboration during the H5N1 avian influenza epidemic that began in 2003 and this materialised in experienced coordination and joint investment in the ‘Tripartite’, recognising ‘a shared responsibility in the management of zoonotic diseases and other threats at the human-animal-environment interface’. In 2010, the Tripartite set out strategic directions for the coordination of global activities to address shared health risks at the human-animal-environmental interface, including in the area of health security and zoonoses prevention and control.^5^ Further to this, in 2017, the Tripartite revisited its joint priorities^6^ and in 2018 a Memorandum of Understanding was signed to confirm support for the collaboration.^7^ In 2021, the Tripartite collaboration expanded with the inclusion of United Nations Environment Program (UNEP), creating a pathway through the Quadripartite Joint Plan of Action for strengthening the environment components of One Health.^8^

Supporting the Quadripartite’s collective role in One Health, in 2020 a multidisciplinary One Health High Level Expert Panel (OHHLEP) was convened to enhance cross-sectoral collaboration. The Quadripartite endorsed OHHLEPs definition, stating that *One Health* is ‘an integrated, unifying approach that aims to sustainably balance and optimise the health of people, animals and ecosystems. This definition recognises the health of humans, domestic and wild animals, plants and the wider environment (including ecosystems) are closely linked and inter-dependent.^9^ The approach mobilises multiple sectors, disciplines and communities at varying levels of society to work together to foster well-being and tackle threats to health and ecosystems, while addressing the collective need for clean water, energy and air, safe and nutritious food, taking action on climate change and contributing to sustainable development’. In addition, OHHLEP developed a theory of change designed to guide OHHLEP’s own work and that of the Quadripartite, providing a framework and key principles, including equity, parity, equilibrium, stewardship and transdisciplinarity, ultimately supporting improved collaboration at all levels of governance.^9^

With this progressive foundation in One Health, the Tripartite has worked alongside global partners to review international frameworks and develop facilitative processes and tools to support governments in strengthening core capacities and effective multisectoral collaboration in preventing, detecting and responding to health risks at the human-animal-environment interface. This includes strengthening countries’ capacity to comply with the WHO International Health Regulations (IHR)^10^ and the internationally adopted WOAH Standards on terrestrial and aquatic animal health and welfare (later referred to as the *Terrestrial and Aquatic Animal Health Codes*).^11^ This collective work has allowed countries to align their sector-specific frameworks for shared goals, clarifying mandates and optimise the results of countries capacity assessments, ultimately providing pragmatic and essential options for improving their preparedness and response capacities and thus, health security.^12^ The Tripartite has developed and supported the use of capacity strengthening tools and programmes, including the IHR-Performance of Veterinary Services (PVS) National Bridging Workshops (NBW)^13^ and the development of the Tripartite Zoonoses Guide (TZG)^14^ and related operational tools (OTs), among other shared efforts, ultimately supporting improved capacity assessment at country level.

**Figure 1:**
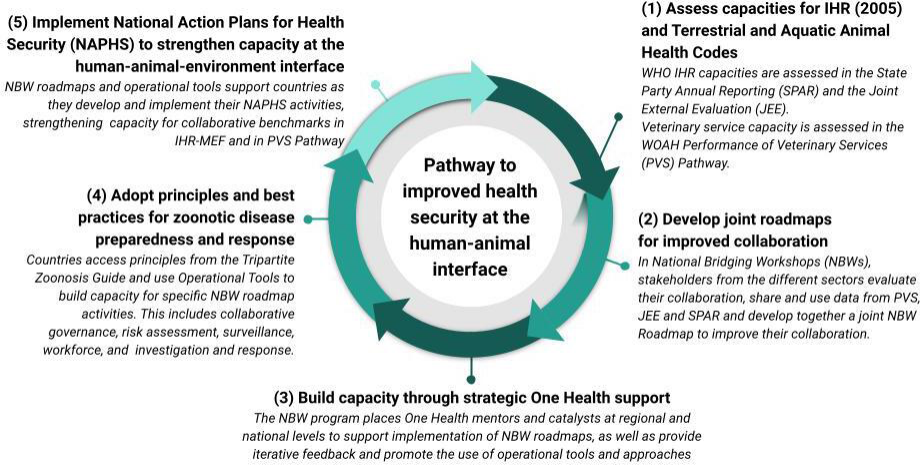
Tripartite pathway for improved health security at the human and animal interface. This pathway enables countries to organise actions and improve capacities in critical technical areas at the human-animal-environment interface. The logical progression allows countries to use IHR-MEF and WOAH PVS Pathway to assess collaborative needs, identify, prioritise and plan corrective measures and then select from various tools and approaches available across the Tripartite and global partners. IHR-MEF, WHO International Health Regulations Monitoring and Evaluation Framework; PVS, Performance of Veterinary Services; WOAH, World Organisation for Animal Health.

As a result of a suit of successive complementary Tripartite activities conducted in countries, a stepwise method has emerged and continues to be updated and improved as new data become available, depicting a pathway (shown in figure 1) enabling countries to organise and prioritise actions for the improvement of their capacities in critical technical areas at the human-animal-environment interface. The pathway follows a logical progression where countries first review the performance of their human and animal health sectors using IHR-Monitoring and Evaluation (MEF) and WOAH PVS Pathway, assess their collaborative needs, identify, prioritise and plan corrective measures through the NBW programme, then select from various tools and approaches outlined in the TZG. This continuum of activities as experienced in several countries ensures that performance evaluation guides capacity strengthening, allowing them to engage effectively across sectors.

Using the WHO IHR-MEF, the WOAH PVS Pathway and FAO Epidemiology Mapping Tool (EMT), Laboratory Mapping Tool (LMT), the Surveillance Evaluation Tool (SET) and other available tools, as appropriate, to review the needs for improved One health coordination between human and animal health sectors

**Figure 2:**
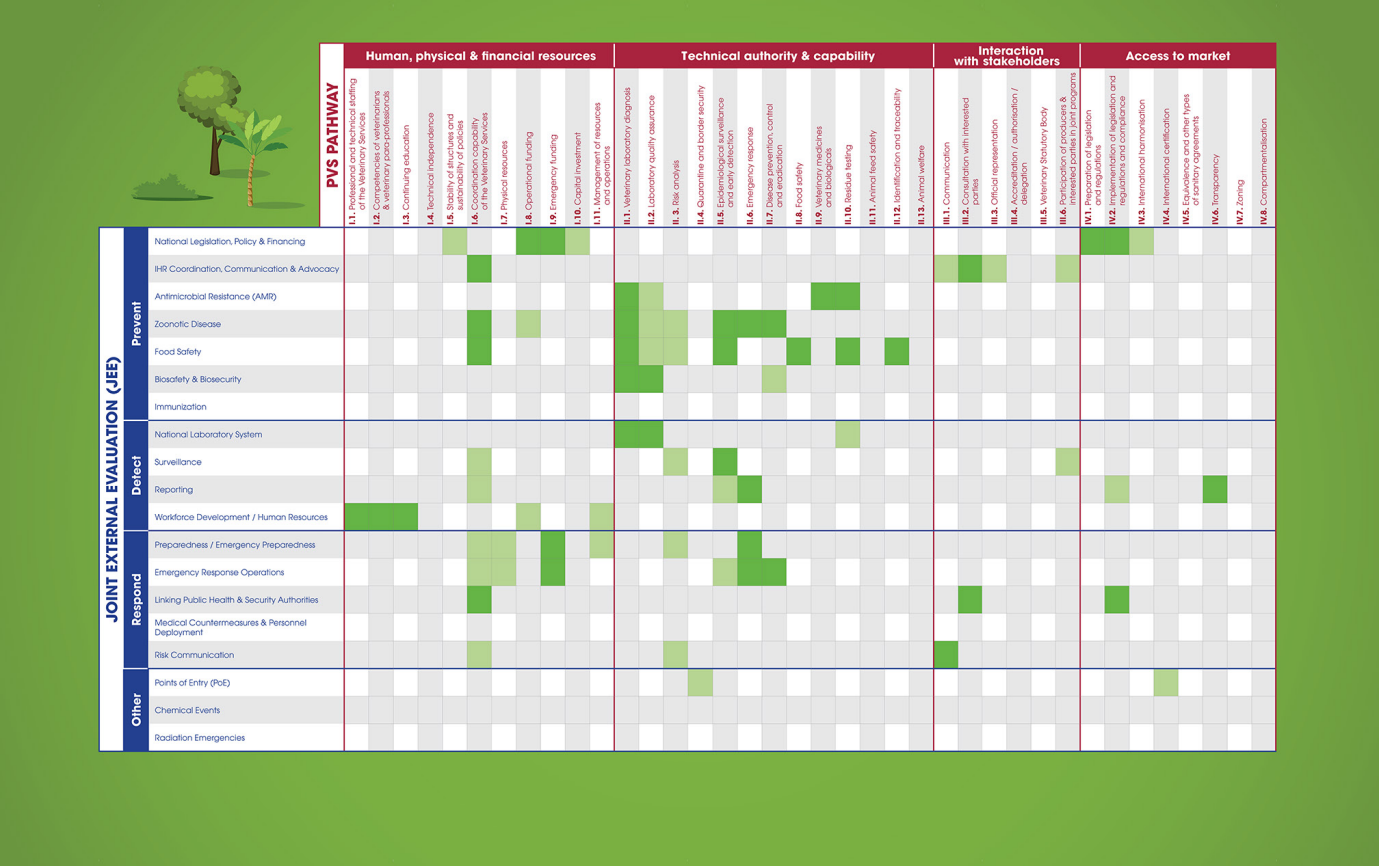
Overlap between IHR-MEF indicators and PVS Pathway critical competencies. IHR-MEF, WHO International Health Regulations Monitoring and Evaluation Framework; PVS, Performance of Veterinary Services.

Historically, international policies (eg, regulations and guidelines), evaluation frameworks and capacity building tools have focused largely on sector-specific needs and mandates. National governments have used the IHR^15^ to align efforts for improved health security as it relates mainly to their health ministries. In turn, countries have used the WOAH’s *Terrestrial* and *Aquatic Animal Health Codes* containing standards for improving animal health through measures for the detection, reporting and control of pathogenic agents as well for preventing their spread.^10 11^ This allows sectors to evaluate their individual sector-specific performance through the IHR-MEF and the PVS Pathway respectively.^15^ The IHR-MEF consists of both the voluntary Joint External Evaluation (JEE) tool and the mandatory States Party Annual Reporting (SPAR) to the IHR secretariat, plus simulation exercises and intra/after action reviews to test these performances.^10^ While these capacity and evaluation frameworks are commonly interpreted through a sectoral lens, there are many important synergies that highlight the necessity of collaborative efforts. For example, the IHR-MEF functions through a whole-of-government approach to address public health events including those arising at the human-animal-environment interface. While the PVS Pathway, the JEE and the SPAR capacity assessments will continue to evolve to meet country needs, in the latest NBW workshops, 19 of the JEE technical areas corresponded with 16 of the WOAH PVS ‘Critical competencies’ (or technical areas of the PVS Tool used for PVS Evaluations^16^ (figure 2). Similarly, the WOAH PVS Tool has a significant component on veterinary public health (including food safety, zoonoses and antimicrobial resistance) and on coordination with other competent authorities. When these assessments are mapped during the NBW workshops, countries can clearly see the overlap and areas of shared priority across the human and animal interfaces. More recent efforts also look to identify areas of priority that similarly support the inclusion of the environment in the NBW programme. Additionally, some more capacity-specific tools such as the FAO SET, the LMT and the EMT evaluate, among other aspects of veterinary capacity, the collaboration of the relevant sectors to jointly detect, prevent, respond and control to health threats at the interface. While not based wholly on the PVS and JEE assessments, these evaluation tools incorporate information gathered through both assessments and generate targeted capacity improvement plans and monitor the implementation of recommendations.

While in many instances these frameworks were created to evaluate respective sectors, as demonstrated in figure 2, they can be used to support multisectoral coordination at country level. By using legal and regulatory frameworks that national professionals are familiar with, collaborative efforts are based on a foundation of ongoing sector-specific work. This ensures that the human and animal health sectors can see value in their sector-driven mandates and extrapolate those efforts to advance a One Health approach.^17^ As an example of this, the Tripartite has seen regional efforts build on the findings of the IHR‐MEF and NBW and developed their regional One Health frameworks such as ‘One Health operational framework for action for the WHO’s Eastern Mediterranean Region (EMRO), focusing on zoonotic diseases’.^18^ This framework specific to EMRO, capitalises on current opportunities in the region and provides countries with a list of practical activities, optimising their resources and strengthening their capabilities to tackle concurrent and future health challenges and jointly achieve sector-specific and collaborative capacities.

## National Bridging Workshop programme provides a first step for planning coordination between human and animal health sectors

The effort to bridge the IHR-MEF and the PVS Pathway resulted in the iterative development and implementation of the NBWs which have to date been conducted in over 35 countries.^13^ These workshops provide the opportunity for the human and animal health sectors to jointly review the results of the IHR MEF (JEE and/or SPAR) and PVS Pathway and to agree on concrete and time-bound activities to fill existing gaps in their coordination for the core functions of the IHR^15^ and fulfilment of the Animal Health Codes.^19 20^ The exercise, conducted with professionals from multiple sectors and operating at various administrative levels in the national systems, results in a jointly developed, detailed, practical and consensual roadmap prioritising 20–30 national activities to improve coordination across ministries.

While the NBW roadmaps are an important step in supporting governments to create shared evidence-based priorities for collaborative engagement, countries have continued to request support for roadmap implementation. Because health efforts are often siloed and frequently sectoral, governments need the support of dedicated persons to promote the implementation of roadmaps and whose priority was to engage sectors in a systems-based approach at national/subnational levels. To that end, in 2020, the Tripartite launched a programme with the objective to support implementation of the NBW roadmaps. This was achieved at a national level by dedicated national consultants (or ‘NBW One Health Catalysts’) hired to serve as critical connectors within government systems and One Health coordinating mechanisms. The NBW One Health catalysts from about 20 countries to date form a Community of Practice supported technically and logistically by Tripartite regional mentors. One of the tasks of the NBW One Health catalysts is to propose and support the use of relevant OTs to facilitate implementation of the NBW roadmap in priority areas, be they developed by the Tripartite and/or partners. These consultants are supported through a community of practice to think strategically about engaging under resourced ministries and linking synergistic tools and approaches.

## Using best practices for zoonotic disease preparedness and response to create operational tools and online trainings that support country implementation of NBW roadmaps

**Figure 3:**
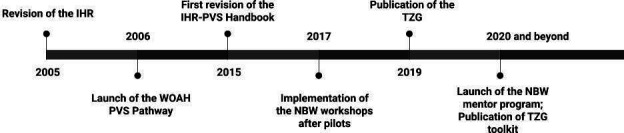
Chronology of tool development associated with the IHR and the PVS Pathway. IHR, WHO International Health Regulations; NBW, National Bridging Workshops; PVS, Performance of Veterinary Services; TZG, Tripartite Zoonoses Guide; WOAH, World Organisation for Animal Health.

As countries mobilised for the development of One Health capacities, including through the NBW roadmap implementation, they requested additional guidance and OTs and resources for strengthening multisectoral collaboration and coordination. A timeline of this progressive development of tools and approaches that support IHR-MEF and PVS pathway is outlined in figure 3. The results of the NBW roadmaps highlighted some of the more challenging technical areas in which national partners need urgent support. In response, the Tripartite gathered over 100 international experts and collected nearly 80 country experiences to develop and publish the 2019 TZG,^14^ an update and expansion to the previous jointly developed guide: the 2008 Tripartite ‘Zoonotic Diseases: A Guide to Establishing Collaboration between Animal and Human Health Sectors at the Country Level’.^21^ The TZG provides guidance and best practices for addressing zoonotic diseases in countries. Developed for use by national staff from all relevant sectors, the TZG has specific technical chapters which are designed to support countries in systematically applying a multisectoral, One Health approach to shared challenges, ultimately identifying gaps in capacity while supporting compliance and strengthening for capacity evaluation frameworks.

To further support countries in implementing key principles outlined in the TZG, a suite of OTs are under development based on the technical chapters of the TZG and will collectively comprise the TZG Toolkit made available across the Tripartite. These include the Joint Risk Assessment OT (JRA OT), the Multisectoral, One Health, Coordination Mechanism OT (MCM OT), the Surveillance and Information Sharing OT (SIS OT) which are published and available in all UN languages and have been used collectively in over 30 country workshops. Additional OTs, such as the Response Preparedness OT (REPREP OT), the workforce development OT (WFD OT) and the Monitoring and Evaluation OT (ME OT), are in development and pilot. Following a similar stepwise approach, each tool includes guidance on how to create an enabling environment, how to conduct the respective activity through technical steps and how to use results across multiple sectors.

## Impacts and lessons learned from pathway implementation at country level

Multiple countries, including Armenia, Kazakhstan and Kenya, have used these collective assessments, tools and approaches to support improved One Health. Even amidst the COVID-19 pandemic, these countries conducted NBWs to first create their NBW roadmaps, highlighting activities that jointly supported improved capacity for IHR MEF and PVS Pathway. In all these countries, one of the roadmap activities suggested the establishment or strengthening of a national One Health mechanism, sometimes referred to as a One Health platform or task force. As a result, countries were able to use the MCM OT to create an action plan for the development of a government One Health mechanism in Armenia and Kazakhstan and the strengthening of the existing Zoonotic Disease Unit in Kenya. This is an example of how bridging the IHR MEF and PVS Pathway through the NBW can lead to shared priorities and collective action. These country examples highlight the importance of country context and flexible implementation of OTs. For example, both Kazakhstan and Armenia had limited One Health engagement at national level and no existing government One Health mechanism. In contrast, Kenya has been supporting government-led One Health collaboration through their Zoonotic Disease Unit for over a decade. The MCM OT was able to be flexibly adapted so that countries with no mechanism could focus on structures, policies and financing for their mechanism and countries with an existing mechanism could focus on activity implementation, capacity strengthening and evaluation, cascading to subnational levels and capacity strengthening and evaluation.

Supported by NBW One Health catalysts, countries have also used the TZG toolkit and partner tools in tandem to conduct activities in their NBW roadmap. For example, Ukraine had recently expressed interest to use the JRA OT for priority zoonotic diseases. Because Ukraine had yet to prioritise zoonotic diseases, the One Health catalyst was instrumental in coordinating the efforts, identifying national facilitators and participants and suggesting the use of the CDC Zoonotic Disease Prioritization Tool first, pairing both tools sequentially. Such engagement contributes to strengthening the Tripartite collaboration with external partners and across all levels, including headquarters, regional and country levels, ultimately supporting improved capacity assessment through IHR-MEF and PVS Pathway.

Implementation of this pathway has not been without unforeseen challenges as highlighted by the COVID-19 pandemic. When countries needed the most support, the Tripartite and external partners were limited in their capacity due to ongoing sanitary situation and global travel restrictions. To support countries, the Tripartite worked to make the principles and best practices outlined in the TZG and OTs freely available through online trainings. For example, the online trainings and a host of additional facilitation support materials allowed for the Tripartite to support hybrid (in-person and virtual) workshops to conduct the JRA OT for priority zoonotic diseases in countries. This has allowed for a focused emphasis on creating flexible online resources and trainings for facilitation of capacity building tools at country level. As of October 2022, over 17 000 learners from all 6 WHO regions have enrolled in the open access online training courses (available from the Tripartite via OpenWHO) for the TZG and the JRA OT, with trainings for additional OTs forthcoming.

Even with challenges brought by the pandemic, impact evaluations for both the NBW and JRA OT, among other Tripartite OTs being developed and piloted, are underway. The post-NBW evaluations from 32 countries have already demonstrated that 98.4% of participants believed the NBW had a positive impact on the collaboration between animal health and human health. As the world continues to be challenged by emerging and re-emerging zoonotic pathogens, these ongoing evaluations have highlighted lessons learnt from the implementation of Tripartite tools and resources and can inform future work of the quadripartite. For example, countries consistently reflect on the need for political will and sustainable resourcing of collaborative activities. As outlined in the NBW results when countries are asked to rate their collaboration on a 1–3 Likert scale for key technical areas, they frequently highlight challenges with collaborative funding, coordinated surveillance and joint risk assessment as well as education and training opportunities for One Health.^13^ It has been noted that punctual ad hoc emergency-based support is not sufficient, and countries are eager to engage in deeper review of their multisectoral operational capacities to ensure appropriate level of preparedness and response for health security threats. An analysis of 22 NBW Roadmaps showed that 20 countries (90.9%) planned for the identification of focal points in relevant sectors at the district/local level for improved coordination, 19 (86.4%) expressed a need to establish a joint information sharing platform and 18 (81.8%) committed to conducting training for joint investigation and response. While Tripartite efforts described in this paper are focused more acutely on the human and animal interface, with the inclusion of UNEP, there is great opportunity to further include the environment sectors into the use of these One Health tools and approaches, strengthening the pathway for health security outlined here. The Quadripartite One Health Global Plan of Action now includes environment perspectives in each of its six action tracks, including one more specifically dedicated to Environment and Health.^8^

## Ongoing and future Quadripartite collaboration for One Health capacity development in countries

The continuum in which countries move forward in their capacity building efforts for One Health, as illustrated previously in figure 1, has evolved through ongoing collaboration across the Tripartite and a willingness to continually improve based on lessons learnt during frontline implementation. As an example, the Tripartite continues to find new avenues for collaboration as seen with the development of field epidemiology competencies in the One Health context and further curricula development and continuing education. By supporting government ministries to align their sector-specific national efforts using data from IHR-MEF and the PVS Pathway, and complementing with FAO evaluations such as EMT, LMT and SET, governments can better envisage a collaborative approach that is founded on existing priorities. Through the NBW Program, countries develop roadmaps that can then be implemented with the support of regional mentors and consultants participating in the One Health Community of Practice who are well positioned to recommend and implement Tripartite OTs to enhance a One Health approach. This systems-based approach offers each country a menu of tools and strategies that can be tailored to meet their national context and identified needs while also contributing to a global vision for improved collaboration at the human-animal interface. The Tripartite to see OTs developed and integrated as one of many options available to countries that can be complemented by tools and approaches of partners from around the globe, including universities, research institutes and government partners.^3 4^ The summative result is a better collaboration for health security and ultimately improved compliance with IHR^15^ and WOAH’s *Terrestrial and Aquatic Animal Health Codes*. In addition, the role of other sectors, including the environment, remains a top priority. Through the inclusion of UNEP in the Quadripartite collaboration, a new focus will be on further strengthening the inclusion of the environment sectors in all aspects of a coordinated and systems-based One Health response in countries. This is being further outlined in the Quadripartite One Health Global Plan of Action and will guide the integration of the environment as a foundational element of the One Health approach.^8^ This iterative, inclusive and developmental approach will allow the Tripartite to continue creating new methods, approaches and evidence-based tools, supporting countries to cultivate a coordinated and systematic One Health approach for existing and emergent health challenges at the human-animal-environment interface, thereby safeguarding global health security for generations to come.

## Data Availability

Data are available in a public, open access repository. Not Applicable.
